# Quantifying Bone Collagen Fingerprint Variation Between Species

**DOI:** 10.1111/1755-0998.14072

**Published:** 2025-01-29

**Authors:** Andrew Baker, Michael Buckley

**Affiliations:** ^1^ Manchester Institute of Biotechnology, School of Natural Sciences University of Manchester Manchester UK

## Abstract

Collagen is the most ubiquitous protein in the animal kingdom and one of the most abundant proteins on Earth. Despite having a relatively repetitive amino acid sequence motif that enables its triple helical structure, in type 1 collagen, that dominates skin and bone, there is enough variation for its increasing use for the biomolecular species identification of animal tissues processed or degraded beyond the amenability of DNA‐based analyses. In recent years, this has been most commonly achieved through the technique of collagen peptide mass fingerprinting (PMF) known as ZooMS (Zooarchaeology by Mass Spectrometry), applied to the analysis of tens of thousands of samples across over one hundred studies in the past decade alone. However, a robust means to quantify variation between these fingerprints remains elusive, despite being increasingly required due to the shift towards a wider range of wild fauna and those that are more distantly related from currently known sequences. This is particularly problematic in fish due to their greater sequence variation. Here we evaluate the quantification of the relative closeness of collagen fingerprints between families using ANOSIM and a modified SIMPER analysis, incorporating relative peak intensity. Our results show a clear correlation between sequence differentiation and statistical distance of PMFs, indicating that the additional complexity of type 1 collagen in fish could directly affect the efficacy of biomolecular techniques such as ZooMS. Furthermore, this multivariate statistical analysis demonstrates that PMFs in fish are substantively more distinct than those of mammalian or amphibian taxa.

## 1 Introduction

One of the most prevalent proteins on Earth (Patino et al. 2002), collagen is particularly abundant in the matrix of bone in chordates and ubiquitous among many other phyla (Boot‐Handford and Tuckwell 2003). As a structural protein, the arrangement and rearrangement of collagen plays a major role in adhesion and tissue growth (Imayama and Braverman 1989; Lee and Boughner 1981; Nuyttens et al. 2011). The fibrillar collagens, found in nearly all phyla, with the notable exceptions of arthropods and nematodes (Boot‐Handford and Tuckwell 2003), are highly conserved (Haq, Ahmed, and Qasim 2019). Generally, they exist as three alpha chains which trimerise to form a triple helix (Pauling and Corey 1951). These chains are formed of repeat peptide sequences of Gly‐Xaa‐Yaa, where the Xaa and Yaa residues can be almost any amino acid but are most commonly proline or hydroxyproline, the latter of which is particularly important for stabilising the triple helical domain through the formation of hydrogen bonds along its axis (Bella and Berman 1996; Bella, Brodsky, and Berman 1995; Engel and Bächinger 2005). This basic template for fibrillar collagen seems to have been established early in metazoan evolution (Zhang and Cohn 2008), with the presence of collagen in Porifera (Boute et al. 1996). There are nearly 30 collagen types known, usually referred to by roman numerals, with types I and III being abundant in most extracellular matrices, and the non‐fibrillar type IV (COL4) being a major component of the basement membrane (Exposito et al. 2010).

In part due to its potential use as a gelatine source (Milovanovic and Hayes 2018), collagen in marine life has been well studied, particularly collagen type I which forms the main component of skin, scales and bone in fish (Duan et al. 2009). It has also been thoroughly exploited in archaeological and palaeontological studies due to its exceptional preservation potential, related to its triple helical structure and high hydroxyproline content (Szpak 2011). However, unlike the collagen of other higher chordates which have triple helices composed of two alpha 1(I) chains and one alpha 2(I) chain, one of the collagen (I) genes of fish has undergone a duplication event resulting in a composition with three genetically distinct chains, where one of the alpha 1(I) chains is referred to as an alpha 3(I) chain (Harvey, Keating, and Buckley 2021). These differences can have an impact on the well‐established biomolecular method of obtaining collagen peptide mass fingerprints for taxonomic identification, also known as Zooarchaeology by Mass Spectroscopy or ZooMS (Buckley et al. 2009). ZooMS relies on enzymatic fragmentation of the protein to provide specific marker peaks unique to a particular taxon. Due to the requirements of the triple helical structure, the peptide sequence for COL1A1 (gene name) is highly conserved, making it an ideal target for such biomolecular techniques across a wide taxonomic range across all vertebrates (e.g., Buckley et al. 2021; Harvey, Daugnora, and Buckley 2018; Buckley and Cheylan 2020; Guiry et al. 2024) with increasing interests in the study of archaeological fish (e.g., Rick et al. 2019; Buckley et al. 2021; Guiry et al. 2020; Hawkins et al. 2022; Winter et al. 2022).

The ZooMS process can readily provide an identification of bone or skin fragments down to genus level, and in some cases to species level (Buckley, Harvey, and Chamberlain 2017). However, in fish, the process of marker selection and differentiation between taxa is much more difficult, in part due to a high level of inconsistency between the fingerprints of the same taxa (Baker, Harvey, and Buckley 2023). There are multiple reasons for this observed inconsistency; in mammalian taxa, where the COL1A1 sequence is highly conserved, differentiation in the collagen sequence leads to more easily identifiable peptide mass fingerprint (PMF) biomarkers (Buckley et al. 2009). In fish, fewer collagen sequences have been published (Song and Wangs 2013) making the confirmation of proposed biologically relevant biomarkers difficult via associated LC–MS/MS sequencing of peptides (e.g., Buckley et al. 2022). However, such exhaustive techniques are highly intensive and may be difficult to implement for large numbers of species. Additionally, due to the higher levels of variation in collagen sequences between fish taxa caused by increased mutation rates relative to other higher chordates (Harvey, Keating, and Buckley 2021), exact and consistent differences are difficult to identify (Buckley et al. 2022). This is exasperated by the relatively poorly described role of post‐translational mutations such as proline hydroxylation that are similar in mass shifts that would otherwise be caused by some amino acid substitutions, such as Alanine‐Serine (difference in mass of 16 Da). This increase in complexity in biomarker identification leads to the necessary supplementation of manual biomolecular methods with computational techniques.

Baker, Harvey, and Buckley (2023) aimed to improve the ease of marker identification through the creation of a Non‐iterative Entropic Dichotomous Marker Identification (NEDMID) tool based on ID3 entropy equations (Quinlan 1982). NEDMID works on the principles of information gain which underscore the ID3 decision tree algorithm, selecting peaks that are likely to be highly descriptive and unique for any given taxa. While this tool appeared highly effective at providing fingerprint markers which are biologically relevant at the family level, at the genus level NEDMID was less effective in some than others. However, it was noted that the relative similarity of fingerprints belonging to the unsuccessfully categorised genera was much lower than that of the fingerprints from the more readily successfully identified genera. For this reason, investigation into those differences was necessary, aiming to identify the peaks that most influence the identifications, and the biological context behind them. Here, we apply a range of statistical processes to quantify the homogeneity of collagen PMFs among a selected group of families and genera, focussing on fish to provide further insight into the complexities of collagen (I) in marine life.

### 1.1 Aims and Objectives

We aim to establish statistical differences between mammalian and fish PMFs using multivariate approaches, and include metrics that are not normally included in analysis. Specifically, intensity data was to be considered alongside *m/z* values for the first time in ZooMS fingerprint analysis, which has proven effective in other types of PMF analysis (Müller et al. 2013; Rossel and Martínez Arbizu 2018). Furthermore, we will assess the actual importance of peaks using not only multivariate statistics as described but also univariate approaches such as the NEDMID algorithm (Baker, Harvey, and Buckley 2023). This holistic data will be compared to phylogenetic information, which will incorporate evolutionary distances and rates of sequence change, to establish authentic substantive differences between taxa in terms of the plausibility and credibility of ZooMS analysis.

The drivers behind PMF variation do not always come from discernible differences within collagen sequences, and there is a wide biomolecular variety resulting from post‐translational modifications (Dai Vu, Gevaert, and de Smet 2018; Witze et al. 2007), which can cause difficulty when attempting to discover new biomarkers for proteomic analysis. Therefore, markers are difficult to predict even when sequence data is available, and biological context can be difficult to apply to potential new markers when comparable sequence fragments are not immediately evident. The statistical interpretations in this report can be used to contextualise PMF variation. For instance, it may provide insight as to the relative importance of post‐translational modifications versus sequence differentiation in determining variation between PMFs within narrow phylogenetic windows, such as inter‐genus comparisons. We aimed to give insight into some of these issues in this analysis.

## 2 Methods and Approaches

### 2.1 Data Acquisition

Data was obtained from a number of different ZooMS datasets, including data from the Caribbean sea (Harvey et al. 2022), the Baltic sea (Harvey, Daugnora, and Buckley 2018), and the Pin Hole cave system in the UK (Buckley, Harvey, and Chamberlain 2017). A breakdown of the taxa from all combined datasets is found in Table S1. Data from the fish datasets was sampled directly from the study by Baker, Harvey, and Buckley (2023), whereas Pin Hole data was taken from the set of samples identified manually, used in the initial database for ML analysis by Gu and Buckley (2018); random samples were taken for use in this study. A single amphibian family was included to provide a reference against the two groups, originally published by Buckley and Cheylan (2020).

Data was then processed with the R (Ihaka and Gentleman 1996; R Core Team 2024) packages MALDIquantForeign (Gibb 2015) and MALDIquant (Gibb and Strimmer 2012) using methods adapted from Gibb and Strimmer (2017), optimised for the above datasets to produce data matrices suitable for multivariate statistical analysis and binary machine learning, both with and without intensity values. Values for data processing and peak picking, such as Half‐Window size, were taken from Baker, Harvey, and Buckley (2023), which was conducted on the same fish datasets. These are shown in Table S2, taken from Baker, Harvey, and Buckley (2023). Further optimisation was required for bespoke analysis, which included estimation of the appropriate SNR for all datasets.

Multivariate analysis was carried out using PRIMER v7 (Clarke and Gorley 2015), while univariate analysis and machine learning was carried out via both statistical methods in RStudio (Ihaka and Gentleman 1996; Navarro 2013; R Core Team 2024) and the NEDMID (Baker, Harvey, and Buckley 2023) Java programmes, available from https://github.com/andrewbkr0/NEDMID.

### 2.2 Analysis of Similarity

Analysis of Similarity (ANOSIM) tests are an excellent multivariate tool for assessing a dataset with numerous variable values (Clarke 1993). Essentially, an ANOSIM tests for statistical relevance in the mean similarity (or dissimilarity) between individuals within and between groups; the null hypothesis in this case is that there is no difference between the groups. Although usually applied to ecological data to compare between communities, we apply it here to collagen PMFs to quantify the relative similarity/dissimilarity between taxa. This yields a good indication of how much disparity exists between the PMFs of taxa belonging to different taxonomic families, and how well conserved they are within those families. This is achieved using the following equation:

R=rB−rWc

When no difference exists between groups, *R* = 0, and the scaling constant c = *n(n‐1)/4*, with *n* representing the total number of samples. This ensures that *R* can only ever be less than or equal to 1, which it would reach if all dissimilarities between groups were larger than all dissimilarities within groups. Multiple permutations were used to calculate *R*.

A Bray–Curtis similarity matrix was constructed following a square root transformation on intensity data. Taxonomic groups were applied to each spectrum as factor data. This matrix was used to inform ANOSIM analysis and non‐metric MDS (nMDS) plot creation.

ANOSIM was applied to collagen PMF data from datasets that cover a range of taxa, including fish, amphibians and mammals. During previous computational analysis, the peaks in each collagen fingerprint were reduced to a binary intensity, a 1 or 0 value, representing the presence or absence of all observed peaks. However, in this case, the intensity values of the fingerprints derived from MALDI‐ToF mass spectrometry were retained, giving us greater insight into the differences between them. The addition of this extra dimension in the data also surpasses some of the limitations of our previously described NEDMID tool; as an algorithm based on the ID3 entropy equations it requires a dataset containing only bivariate features that is not the case for multivariate statistical approaches such as ANOSIM.

### 2.3 Identification of Biomarkers

Similarity Percentage breakdowns (SIMPER) provide two indications (Clarke 1993). First, they can be informative of which features of a dataset contribute to the dissimilarity observed between groups. Second, they can be informative of which features of a dataset can be best described as typical for a particular group. SIMPER analyses determine the contribution of a feature *f* by taking its average over all pairs of samples within a group. In essence, the higher the abundance of a feature within a group, the greater its contribution; standard deviation is also taken into account. A more complete description of the SIMPER approach can be found in Clarke (1993). In our analysis of collagen fingerprints, the ‘group’ refers to the 11 families of fish analysed within the combined dataset, and the features described are the peaks within each fingerprint. It should also be noted that while SIMPER typically handles abundance values of ecological data, we instead apply it here to the intensity values of the observed MALDI peaks. In this report, we compare the output of SIMPER analysis with NEDMID results, to establish whether the peaks that cause statistical differences between taxa are also the peaks identified as biomarkers; we will also assess pairs of taxa that NEDMID frequently misidentifies. NEDMID has been shown to be highly effective at identifying the markers of certain taxa to family level, and subsequently utilise those markers in the identification of new spectra. However, the accuracy of the tool dropped when applied to the genus level. This was thought to be due to a high degree of homogeneity in collagen between fish taxa.

Conducting SIMPER analysis in PRIMER (Untergasser et al. 2012) also provides us with the average similarity of groups, as well as the average dissimilarity between groups, alongside peak contributions to characterisation and differentiation. This value is obtained from mean values of pairwise Bray–Curtis dissimilarity comparisons.

Given the importance of contextualisation in biomarker selection, peaks returned by SIMPER analysis as potentially important were removed if they were not thought to be biologically relevant, such as those with an *m/z* value below 900, or the frequently observed matrix peak at *m/z* 1060.1 (e.g., Harris et al. 2002); these can be readily identified through their decimal value, given most will be close to 0.0 or 0.1, where peptides in this *m/z* range are typically half a Dalton heavier.

### 2.4 Primary Sequence Analysis

Whilst multivariate analyses can contribute to the identification of statistically important peaks, it cannot provide any biological context, for which it would be useful to incorporate peptide sequence data where possible. To do this, we cross‐referenced important differentiator peaks identified through SIMPER analysis with retrievable collagen sequences of the relevant taxa to determine the region responsible for this peak. We then compared this to other sequences to identify distinguishing peptide regions. Amino acid sequences were obtained using ENSEMBL genome browser (Howe et al. 2021) and subsequent protein BLAST searches using NCBI (Pruitt, Tatusova, and Maglott 2005). Only COL1A1 sequences were considered from Actinopterygii taxa, which corresponded to the families present in the datasets described above, enabling direct comparison of PMF and sequence data. In the case of Siluridae and Percidae, multiple sequences were found from the same family and were included to provide information on the relative closeness of sequences within families and between them.

Sequences were aligned in BioEdit (Hall, Biosciences, and Carlsbad 2011) and two measures were taken; Count Difference and Sequence Identity (Girgis, James, and Luczak 2021). These results, given pairwise between taxa, were then compared to both the R statistic and the predicted number of amino acid changes taken from the rate of change described in Harvey, Keating, and Buckley (2021); COL1A1 = 0.7, COL1A2 = 0.9, COL1A3 = 1.0. Evolutionary distances are expressed as median estimates of time since divergence, obtained from TimeTree (Kumar et al. 2022) using values for analysis. Rates of amino acid change as above were applied to the evolutionary distances to obtain predicted change rates.

## 3 Results

### 3.1 Measuring Similarity

The ANOSIM results for the fish fingerprints at the family level (Table 1) found an overall R statistic of 0.809, which indicates a high level of distance between the families. Most pairs of families showed differences above 0.8, with some particularly low‐value exceptions in Haemulidae versus Lutjanidae (*R* = 0.389), Haemulidae versus Scaridae (*R* = 0.381), Scaridae versus Lutjanidae (*R* = 0.290), Labridae versus Scaridae (*R* = 0.202) and Lutjanidae versus Labridae (*R* = 0.346).

**TABLE 1:** ANOSIM results for fish families, obtained following a square root transformation on a matrix of spectra including intensity information via a Bray–Curtis similarity matrix (Bray and Curtis 1957). An overall significance level of 0.1% indicates a highly significant result (*p* < 0.01). All pairwise comparisons were also found to be significant (*p* < 0.05).

Groups	R stat	Sig. level %	Possible perms	Actual perms	No. > = Observed
Cyprinidae, Esocidae	0.719	0.1	Very large	999	0
Cyprinidae, Haemulidae	1	0.1	170,544	999	0
Cyprinidae, Labridae	0.997	0.1	170,544	999	0
Cyprinidae, Lutjanidae	0.947	0.1	3.01E+08	999	0
Cyprinidae, Percidae	0.808	0.1	1.04E+09	999	0
Cyprinidae, Scaridae	0.82	0.1	Very large	999	0
Cyprinidae, Scombridae	0.996	0.1	Very large	999	0
Cyprinidae, Scophthalmidae	0.989	0.1	3,268,760	999	0
Cyprinidae, Serranidae	0.98	0.1	Very large	999	0
Cyprinidae, Siluridae	0.973	0.1	3,268,760	999	0
Esocidae, Haemulidae	0.987	0.1	888,030	999	0
Esocidae, Labridae	0.941	0.1	888,030	999	0
Esocidae, Lutjanidae	0.94	0.1	Very large	999	0
Esocidae, Percidae	0.73	0.1	Very large	999	0
Esocidae, Scaridae	0.927	0.1	Very large	999	0
Esocidae, Scombridae	0.959	0.1	Very large	999	0
Esocidae, Scophthalmidae	0.784	0.1	30,045,015	999	0
Esocidae, Serranidae	0.987	0.1	Very large	999	0
Esocidae, Siluridae	0.807	0.1	30,045,015	999	0
Haemulidae, Labridae	0.772	0.1	1716	999	0
Haemulidae, Lutjanidae	0.389	0.1	245,157	999	0
Haemulidae, Percidae	0.981	0.1	480,700	999	0
Haemulidae, Scaridae	0.381	0.3	22,481,940	999	2
Haemulidae, Scombridae	0.922	0.1	2,629,575	999	0
Haemulidae, Scophthalmidae	0.999	0.1	19,448	999	0
Haemulidae, Serranidae	0.722	0.1	2.03E+08	999	0
Haemulidae, Siluridae	1	0.1	19,448	999	0
Labridae, Lutjanidae	0.346	0.5	245,157	999	4
Labridae, Percidae	0.951	0.1	480,700	999	0
Labridae, Scaridae	0.202	4.5	22,481,940	999	44
Labridae, Scombridae	0.806	0.1	2,629,575	999	0
Labridae, Scophthalmidae	0.998	0.1	19,448	999	0
Labridae, Serranidae	0.558	0.1	2.03E+08	999	0
Labridae, Siluridae	1	0.1	19,448	999	0
Lutjanidae, Percidae	0.902	0.1	Very large	999	0
Lutjanidae, Scaridae	0.29	0.1	Very large	999	0
Lutjanidae, Scombridae	0.703	0.1	Very large	999	0
Lutjanidae, Scophthalmidae	0.954	0.1	5,311,735	999	0
Lutjanidae, Serranidae	0.6	0.1	Very large	999	0
Lutjanidae, Siluridae	0.992	0.1	5,311,735	999	0
Percidae, Scaridae	0.883	0.1	Very large	999	0
Percidae, Scombridae	0.982	0.1	Very large	999	0
Percidae, Scophthalmidae	0.855	0.1	13,123,110	999	0
Percidae, Serranidae	0.985	0.1	Very large	999	0
Percidae, Siluridae	0.924	0.1	13,123,110	999	0
Scaridae, Scombridae	0.611	0.1	Very large	999	0
Scaridae, Scophthalmidae	0.949	0.1	Very large	999	0
Scaridae, Serranidae	0.47	0.1	Very large	999	0
Scaridae, Siluridae	0.97	0.1	Very large	999	0
Scombridae, Scophthalmidae	1	0.1	1.31E+08	999	0
Scombridae, Serranidae	0.78	0.1	Very large	999	0
Scombridae, Siluridae	1	0.1	1.31E+08	999	0
Scophthalmidae, Serranidae	0.991	0.1	Very large	999	0
Scophthalmidae, Siluridae	0.993	0.1	92,378	999	0
Serranidae, Siluridae	0.993	0.1	Very large	999	0

**FIGURE 1:**
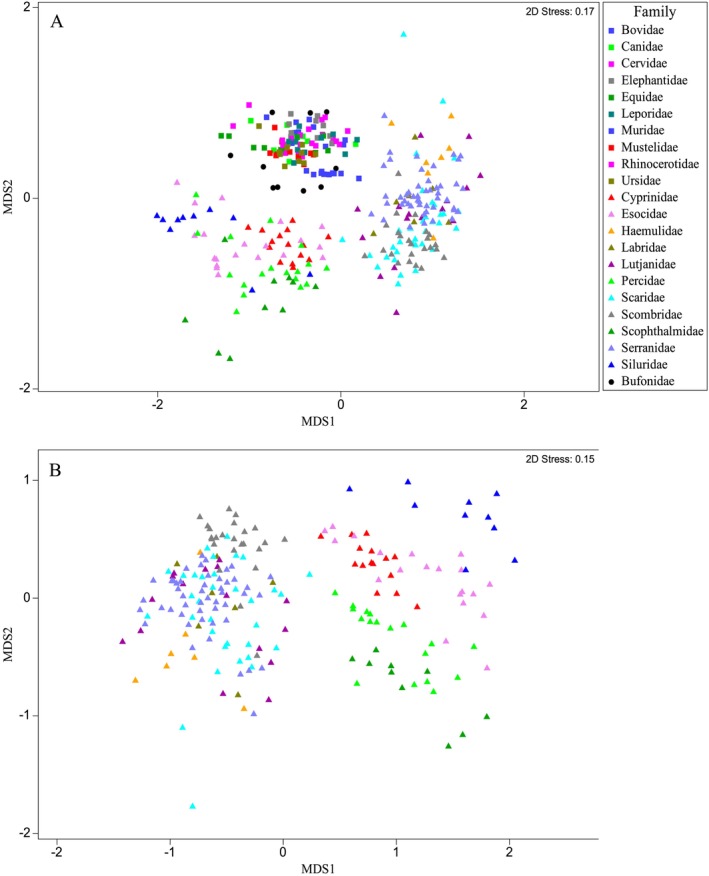
(A) A non‐metric MDS plot demonstrating the relative similarity of collagen fingerprints belonging to families of fish (triangle), mammals (square) and amphibians (circle) informed via a Bray–Curtis similarity matrix (Bray and Curtis 1957), using MALDI spectra data with intensity values included. (B) A non‐ metric MDS plot demonstrating the relative similarity of collagen fingerprints belonging to 11 families of fish. Informed via a Bray–Curtis similarity matrix, using MALDI spectra data with intensity values included.

Spectra from families of mammals, amphibians and fish were compared using Multi‐Dimensional Scaling (MDS) to highlight the relative statistical distance between PMFs of different families from these three taxonomic groups (Figure 1A). The mammal PMFs clustered well together, whereas those from fish families appeared highly disparate. Similarly, the amphibian PMFs are also relatively well clustered, although two different genera may be represented here (Buckley and Cheylan 2020), sitting between the mammals and the fish in Figure 1A. This mirrors their phylogenetic relationship, although this is likely an incidental or indirect relationship (Meyer and Zardoya 2003). Figure 1B shows that the 11 fish families cluster well together, supporting the high ANOSIM scores observed (Table 1). However, there are some families with notably high disparity, such as Lutjanidae or Scaridae, with individuals from these two families forming separate clusters—this reflects the particularly low ANOSIM score between the two groups. This does not appear to reflect individuals from separate studies.

**FIGURE 2:**
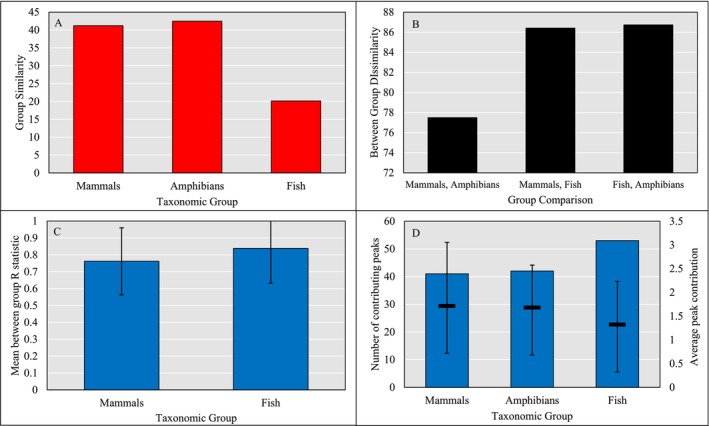
Bar charts showing the (A) Average Similarity and (B) Dissimilarity per taxonomic group, following a square root transformation on a matrix of spectra including intensity information obtained from Bray‐Curtis comparisons (Bray and Curtis 1957) alongside SIMPER analysis, (C) Mean R statistic obtained between families of fish and of mammal (amphibian data not included as only one family present) from ANOSIM analysis of a Bray–Curtis similarity matrix and (D) Number of peaks forming the top 70% of contributors to group similarity (from SIMPER analysis) as bars, as well as the mean contribution of those peaks; error bars show ± standard deviation.

**FIGURE 3:**
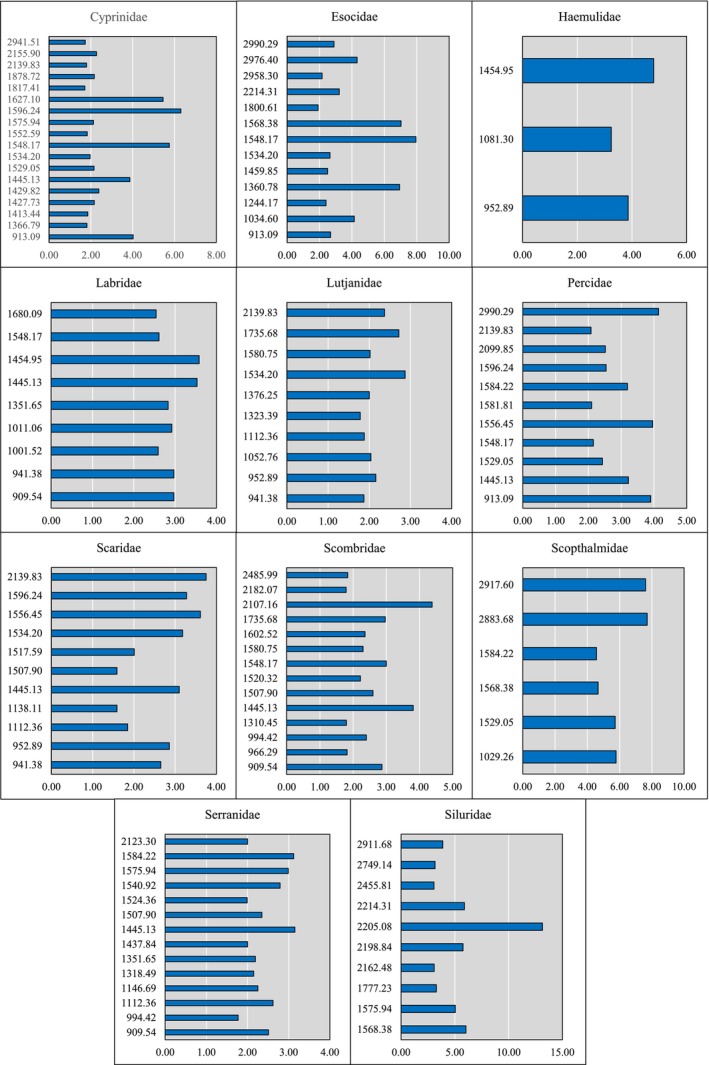
Bar charts showing the relative importance of peaks obtained from SIMPER analysis of a Bray–Curtis similarity (Bray and Curtis 1957) matrix produced from each family of fish. Only peaks determined to contribute to the top 50% of total importance for a taxa's similarity score are included. PMFs underwent a square root transformation after being arranged in a matrix of spectra including intensity information and peaks that were determined to be from likely non‐biological sources, such as peaks caused by the crystallising matrix, were removed from the analysis.

ANOSIM results were also obtained for mammal and amphibian families performed alongside SIMPER analysis for all three groups (Figure 2). We found that the group similarity score was considerably lower in fish (20.13) than in mammals (41.21) or amphibians (42.46). Similarly, the average dissimilarity score was higher when both mammals and amphibians were compared to fish (86.41 and 86.73, respectively) than when mammals and amphibians were compared directly (77.50). Furthermore, the SIMPER analysis conducted when the dataset was split into mammals, fish and amphibians without familial classifications, revealed that fish yielded a higher number of peaks contributing to the top 70% similarity score (53) than mammals (41) and amphibians (42), as well as a lower mean contribution towards that score per peak (1.32, 1.72 and 1.68, respectively). Therefore, this analysis supports the hypothesis that fish PMFs are statistically different from other higher chordate taxa. Figure 3 also shows that the mean R statistic from ANOSIM between families of mammals is significantly lower (per the results of a two‐sample t‐test) than between fish families (0.76 ± 0.20 and 0.84 ± 0.21, respectively).

### 3.2 Identifying Peptide Markers for Species Determination

Two approaches were utilised for the identification of markers. Firstly, the multivariate analysis described above, namely the SIMPER analysis (Figure 3), which revealed that several markers were present that could be descriptive of multiple taxa; for example, the peak at *m/z* 1548.17 is an important indicator peak for PMFs of 5 of the 11 families. However, there is notable variation in the number of markers present in the top 50% of importance, with some families having only 3 markers (Haemulidae), and others having many more.

**FIGURE 4:**
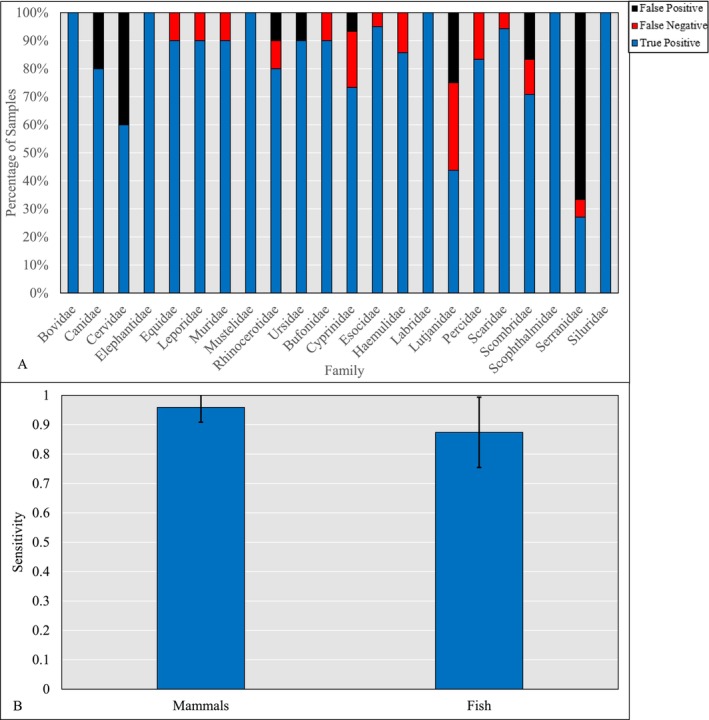
Bar charts showing (A) the proportion of PMFs from each family for which NEDMID returned a true positive (blue), false positive (black) and false negative (red) result, performed on a matrix with intensity values reduced to a binary value indicating the presence or absence of each peak, and (B) the mean sensitivity of the NEDMID tool regarding families belonging to fish, mammal and amphibian taxonomic groups.

The data for mammals, amphibians and fish was also analysed using the NEDMID algorithm (Baker, Harvey, and Buckley 2023) finding that certain mammal families, such as Cervidae had a high false positive rate (frequently classified as Bovidae), while fish tended to have a higher false negative rate (Figure 4), with the notable exception of Serranidae which was misclassified often as Scaridae. However, a higher mean sensitivity was observed in mammals (0.959 ± 0.050) than in fish (0.874 ± 0.119), although the results of a Welch two sample t‐test returned a *p*‐value marginally above the typical threshold of significance (*p* = 0.0597).

### 3.3 Sequence Analysis

**FIGURE 5:**
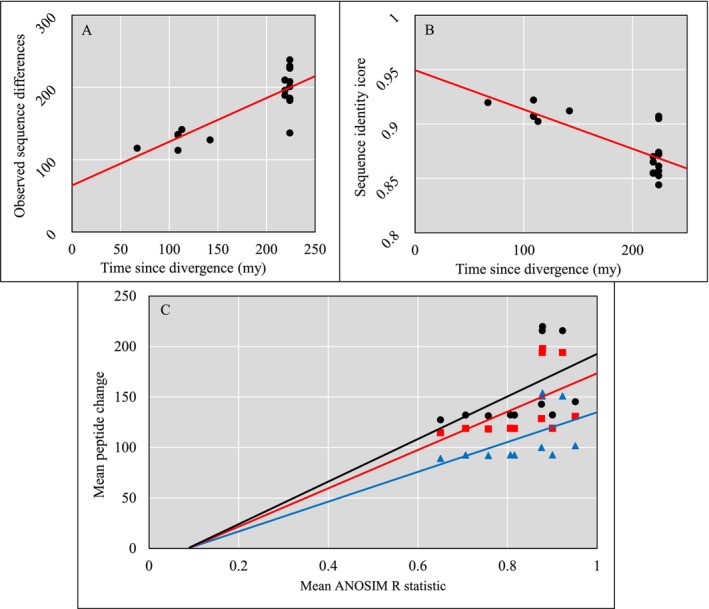
Scatter plots showing comparisons between families of fish PMFs, sequence data and time since evolutionary divergence. (A) Time since divergence in millions of years (mya) against the mean observed differences in nucleotide sequence, where R2 = 0.688 and the correlation coefficient was 0.829. (B) Time since divergence against the mean SIS of compared sequences, where R2 = 0.6037 and the correlation coefficient was −0.777. (C) Scatter plot showing mean R statistic from ANOSIM analysis for each family of fish when compared to other families of fish, against the mean predicted peptide change for COL1A1 (black), COL1A2 (red) and COL1A3 (blue); the R2 value for all three trend lines = 0.260, with a correlation coefficient of 0.510.

The evolutionary distances (ED) expressed as median divergence time estimates obtained from TimeTree show a positive correlation with observed sequence differences, as well as a corresponding negative correlation with sequence identity score, shown in Figure 5A,B, respectively.

Furthermore, the relationship between the R statistic and number of amino acid substitutions is seen in Figure 5C to demonstrate a positive correlation with a coefficient of 0.510, indicating a relationship between sequence dissimilarity and collagen fingerprint dissimilarity.

**FIGURE 6:**
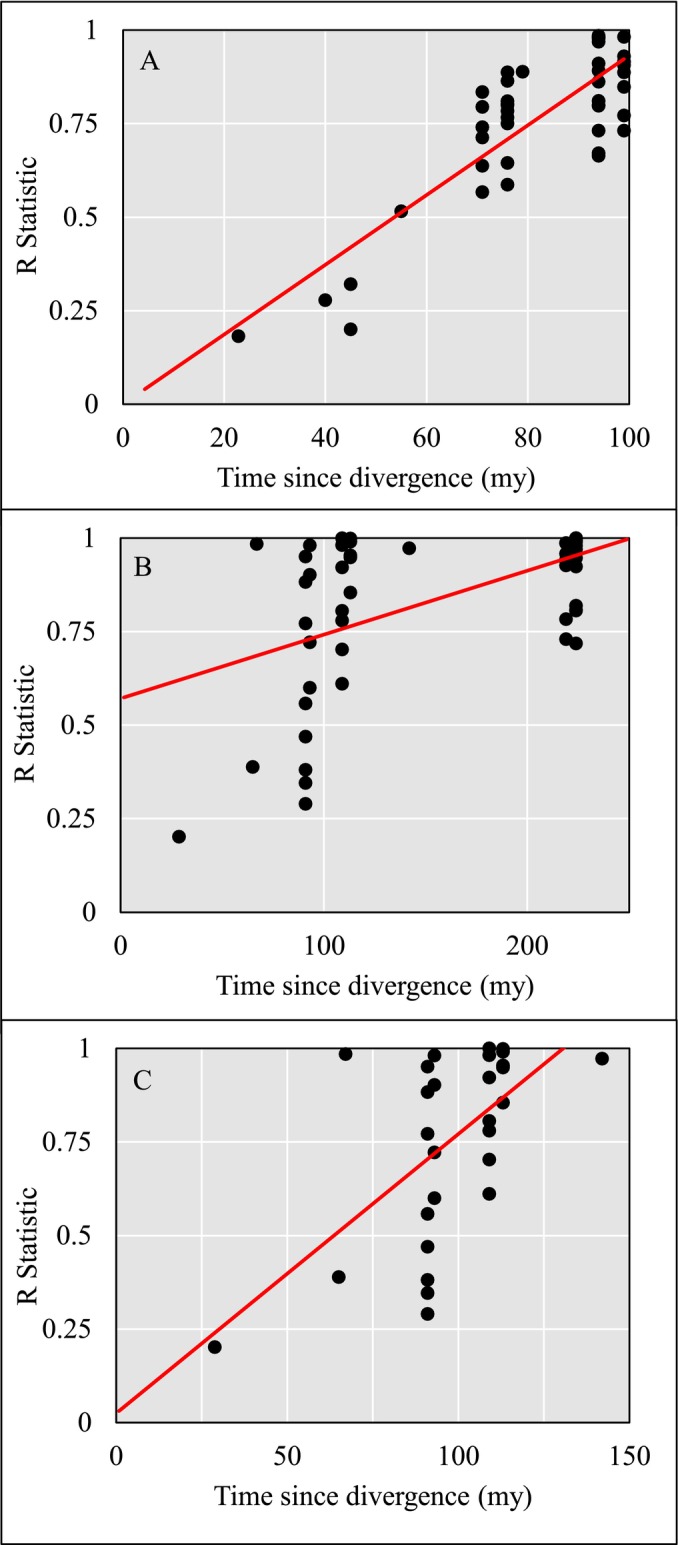
Plots showing the correlation between divergence time in millions of years and the R statistic obtained from pairwise ANOSIM tests. R statistics approaching 1 indicate high levels of dissimilarity between PMFs of the given taxa. (A) Mammals: R2 = 0.707, correlation coefficient = 0.841, (B) fish: R2 = 0.278, correlation coefficient = 0.527 and (C) fish following the exclusion of data points with a divergence time > 150 Mya: R2 = 0.363, correlation coefficient = 0.603.

The R statistic was also positively correlated with ED (Figure 6), in both mammals (R2 = 0.707, correlation coefficient = 0.841) and in fish (R2 = 0.278, correlation coefficient = 0.527). In the fish model (Figure 6B), a banding can be observed with a group of divergence times > 200 Mya; it was initially speculated that this difference could be due to the inclusion of fish from two disparate datasets (Caribbean and Baltic Sea), however, closer analysis indicated that the higher grouping was caused by the presence of a few families which diverged much earlier, including Esocidae, Cyprinidae and Siluridae.

This highlighted a difference between the two taxa; mammals first emerged around 200 Mya, with placental mammals appearing ~80 Mya (Álvarez‐Carretero et al. 2022), whereas Actinopterygii emerged around 350 Mya, with Teleosts diverging > 200 Mya (Harvey, Keating, and Buckley 2021). To account for this potential disparity, a linear model was fitted on fish data following exclusion of any ED > 150 Mya (Figure 6C). This curated fish selection, representing comparisons between families with similar phylogenetic closeness to mammals, showed an increased R2 of 0.363, with an increased correlation coefficient of 0.603.

## 4 Discussion

### 4.1 Collagen Fingerprint Similarity

Previous approaches to the challenges of fish collagen identification using PMFs have typically involved intensive manual analysis Harvey, Daugnora, and Buckley (2018); Harvey, Keating, and Buckley (2021, 2011), with recent attempts to use machine learning approaches (Baker, Harvey, and Buckley 2023; Gu and Buckley 2018) to complement advances in high‐throughput analytical approaches yielding much greater numbers of spectra to be identified (Buckley et al. 2016). However, these have most commonly relied on only the presence/absence of peaks within a spectrum, even those using statistical approaches such as PLSR (Richter et al. 2011). Yet including intensity values in the analysis, as attempted in this study, could give us a much greater insight into the fundamental principles of PMF identification.

First, we observed a significant difference in PMF variance of fish taxa when compared with other higher chordates; mammalian families, although somewhat distinct from one another, were tightly clustered with very little relative distance between the PMFs (Figure 1A), whereas, in contrast, fish families were relatively spread out, showing much higher levels of disparity. When non‐fish taxa were excluded (Figure 1B), we see that although some fish families such as Scophthalmidae or Scombridae are well clustered, other families such as Lutjanidae are not. There are also several examples of families with a small group of individuals that appear to be clustered separately, for instance Scaridae and Haemulidae. This suggests that there is not only an overall disparity between fish PMFs, causing a lack of consistent biomarkers, but also an overlap between taxa causing confusion during biomarker identification. Figure 3 makes this apparent, with a number of peaks appearing to be characteristic markers of PMFs from a number of families during SIMPER analysis. While this is interesting statistically, it also makes practical identification extremely difficult, particularly when we consider that a number of those families only have a handful of markers identified (Buckley et al. 2022, 2021; Harvey, Daugnora, and Buckley 2018; Harvey, Keating, and Buckley 2021; Harvey et al. 2022; Richter et al. 2020, 2011).

Bray‐Curtis comparisons identified group similarity to be significantly higher in mammals and amphibians (Figure 2), suggesting that they may be identifiable using a small number of peaks, which remain relatively consistent through taxonomic levels; the R statistic between mammal families from ANOSIM was observed to be significantly lower than between fish families (Figure 2C). In contrast to mammals and amphibians, fish PMFs were too disparate to be analysed in the same way, needing a higher number of total peaks to characterise the whole group (Figure 2D). Indeed, this is precisely what we observed when analysing fish PMFs; families could not be adequately described by a consistent group of markers for identification to take place to a sufficient taxonomic level, as is normally the case for ZooMS (Bouchard et al. 2020; Buckley et al. 2022, 2021; Morin et al. 2023; Sjögren et al. 2023). Markers which may define one group of Scaridae, for example, do not describe another group of Scaridae.

However, to work out the cause of this, we must first consider the possibility that the original identification of these spectra could have been incorrect. While this may explain a single outlier in a family group, as we see with Esocidae for example, it would not explain why large groups of data were statistically distinct, despite belonging to the same family. One possible reason for this could be that statistically distinct groups belong to the same family but come from different genera. However, this appears to be incongruent with our results (Figures S1 and S2), indicating that sequences from within the same family are considerably more similar to each other than to other families. A more likely explanation is that while there may be certain marker peaks which could be used to define a group of PMFs from a family, there are other peaks which mask the importance of this from distance‐based statistics such as the ANOSIM R statistic. Therefore, relative statistical closeness cannot readily be used alone to assess the differences between PMFs.

### 4.2 Biomarker Identification

As mentioned above, this study includes intensity data in a multivariate approach to PMF analysis. We found that several markers for each family of fish could be identified through SIMPER (Figure 3), not only using their presence across all PMFs from that family but also the similarity of their intensities. NEDMID was also applied to this dataset (Figure 4), showing that although mammal families such as Ursidae have a high rate of false positive identification, fish tend to have a higher false negative rate. This tendency to return ‘Unclassified’ results rather than a false positive is encouraging, particularly given the results observed in Baker, Harvey, and Buckley (2023), which seemed to indicate a tendency to misclassify data from taxa with smaller evolutionary distances, such as Ursidae to Canidae.

The higher propensity of NEDMID to misidentify fish as false negatives also supports the hypothesis that PMF variation is greater in fish. This suggests more robust PMF biomarkers in mammals, likely linked to the higher variance in fish even among individuals within the same family.

### 4.3 Sequence Metrics

It is clear that there is a relationship between the collagen sequences and the observed statistical differences in PMFs (Figure 5C); the ANOSIM R statistic is a good indicator of multivariate statistical difference between the spectra, while the predicted amino acid change informs us not only of a sequential difference but also of an evolutionary distance between families. This link is demonstrated through the correlations in Figure 5. As fish taxa diverge over time, their sequences become more dissimilar, and this has an impact on PMF dissimilarity. This clear linear relationship has not been observed before but does support the conclusion that evolutionary distance directly relates to overall differences in the PMF.

When this relationship is compared directly, we see a positive correlation emerging between time since divergence and PMF dissimilarity. This is shown for both mammals and fish in Figure 6. Given the more significant correlations observed in mammals, even when the fish dataset is reduced to a similar evolutionary timescale as mammalian divergence (Figure 6C), we can determine that time since evolutionary divergence is a more significant factor in mammal PMF divergence, suggesting a relationship between evolutionary ‘closeness’ and PMF similarity which simply does not exist in fish. This may explain the previously indicated difficulties in assessing fish through ZooMS compared to other higher chordates (Baker, Harvey, and Buckley 2023).

We should also consider the role of other factors which may be working alongside sequence divergence; this study does not include the rate or effect of post‐translational modification, physico‐chemical differences in fish bone which may affect the ZooMS process, or environmental factors. However, the discovery of the impact of evolutionary distance on PMF variability underscores the previously observed challenges in categorising fish taxa using ZooMS.

Our study supports the approximate rates of change described previously (Harvey, Keating, and Buckley 2021), showing a direct correlation between the observed and predicted amino acid changes using the observed differences between sequences (Figure 5), enabling inferences with a high degree of confidence based on these findings. This is not only for COL1A1, represented in the sequences here, but also for COL1A2 and COL1A3 as the different rates of change across the three collagen alpha chains correspond to correlations with the R statistic (Figure 5C). It should be noted that the biomarkers present in the PMFs are not restricted to COL1A1—the ZooMS process is non‐selective at this level. Therefore, where slight anomalies appear in the data between what we expect to see based on sequence data and what is observed in the multivariate analysis (based on PMFs), we must consider that this discrepancy could be due to the inclusion of other strands with different sequences and therefore potentially different mass values in the PMF data.

## 5 Conclusions

Utilising a multivariate approach alongside other quantitative measures has revealed several interesting facets to PMF analysis in fish taxa. Fish have long been described as difficult to apply ZooMS analysis to for the purposes of species identification; there are fewer published sequences (Song and Wangs 2013) and the higher degree of variation in collagen (I) sequences makes biomarkers difficult to distinguish (Richter et al. 2020). The multivariate results support this; fish have a higher within group R statistic from ANOSIM, and a drastically lower group similarity from SIMPER. Therefore, we are finally able to quantify the degree of difference between fish PMFs and other, more applicable chordate taxa. The reasons for this difference are difficult to quantify, but it is clear that it begins at the sequence level, even among the highly conserved COL1A1 sequence. This may be why it seems that although fish are disparate as a taxonomic group in terms of PMF similarity, there is still a significant crossover between taxa, which further compounds the issue of attempting biomarker identification at lower taxonomic levels. Furthermore, the results shown in Figure 6 may indicate that one issue with ZooMS on ray‐finned fish, which is less prevalent in other higher chordates, is the relatively large amount of time since many taxa diverged from one another, leading to enormous divergence times with many opportunities for amino acid substitution and its consequent effects on the analysed PMFs.

The results from NEDMID biomarker identification and subsequent PMF classification showed a higher sensitivity in mammals, although fish display a tendency towards false negative results rather than false positive, with the notable exception of the Serranidae. This suggests that although the problem of biomarker identification in fish PMFs is prevalent, there may be hope for reducing this issue in the future, allowing for simple automated identification of reliable and robust marker peaks. This should involve the inclusion of a variety of spectra in the machine learning matrix provided to NEDMID, which can then effectively use the principles of entropy it relies on to determine the true importance of peaks in a wider context; when restricted to only fish PMFs, it may struggle to discern biomarkers from irrelevant background noise. Based on the findings of this study, it may also be prudent to include multivariate analysis in future biomarker identification protocols, to identify distinctive and characteristic peaks. Given the relative ease with which these methods can be applied to large datasets, it is feasible that this type of analysis could form a part of future ZooMS studies in all higher chordates, adding a further tool for biomarker identification. Peaks identified in this manner could be added to reference databases, although it should be noted that their importance is relative only to the dataset in which they are contextualised.

## Conflicts of Interest

The authors declare no conflicts of interest.

## Supporting information


Data S1.

## Data Availability

The authors confirm that all data underlying the findings are fully available without restriction. Software is available on our GitHub repository: https://github.com/andrewbkr0/NEDMID and data is available at10.6084/m9.figshare.27045925, whereas the data could already be found at https://figshare.com/articles/dataset/Ancient_Caribbean_Fish_Collagen_Fingerprints_and_Sequence_Files/20123150 for the Caribbean fish, https://figshare.com/s/0dfe16e51890e61e0706 and https://figshare.com/s/ee95a3c638d1de2def12 for the 10% Fraction data and 50% Fraction data for the Baltic Sea fish data, and 10.6084/m9.figshare.27045925 for pre‐existing Pin Hole cave data, both mammalian and amphibian, made publicly made available for this study.
