# Supplementary material for: Quantifying Bone Collagen Fingerprint Variation Between Species
Source: Mol Ecol Resour. 2025 Jan 29;25(4):e14072. doi: 10.1111/1755-0998.14072 (PMC11969642; doi:10.1111/1755-0998.14072)
Supplement: Supplementary file 1 — Data S1. [file MEN-25-e14072-s001.pdf]

MOLECULAR ECOLOGY RESOURCES

**Supplemental Information for:**

# Quantifying bone collagen fingerprint variation between species

**Andrew Baker, Michael Buckley**

**Table of Contents:**


| | |
|---|---|
| ***Supplementary Figure S1*** | Page 1 |
| ***Supplementary Figure S2*** | Page 2 |
| ***Supplementary Table S1*** | Page 2-3 |
| ***Supplementary Table S2*** | Page 3 |


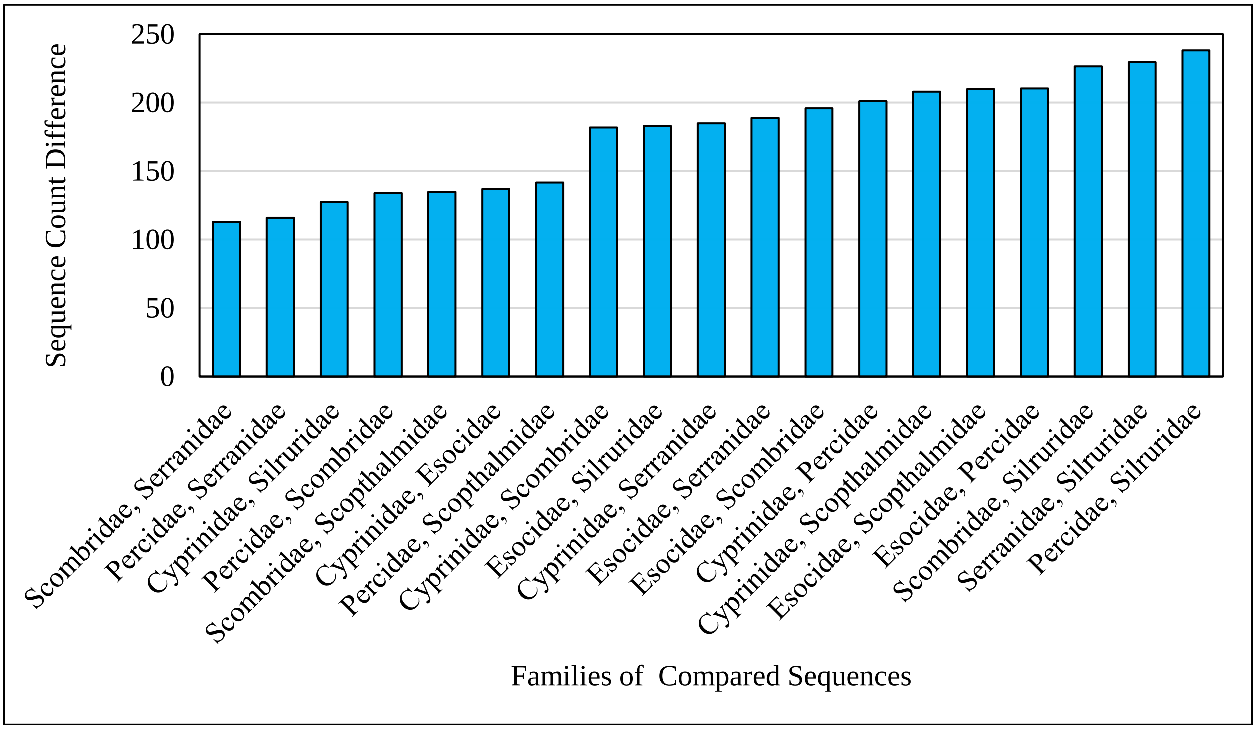


***Supplementary Figure S1 - Displaying the SCD values obtained through pairwise comparison of sequences drawn from an NCBI blast search. Sequences were selected where supporting PMF data was available within the datasets utilised in this study. Taxa information for each sequence is given to the family level.***

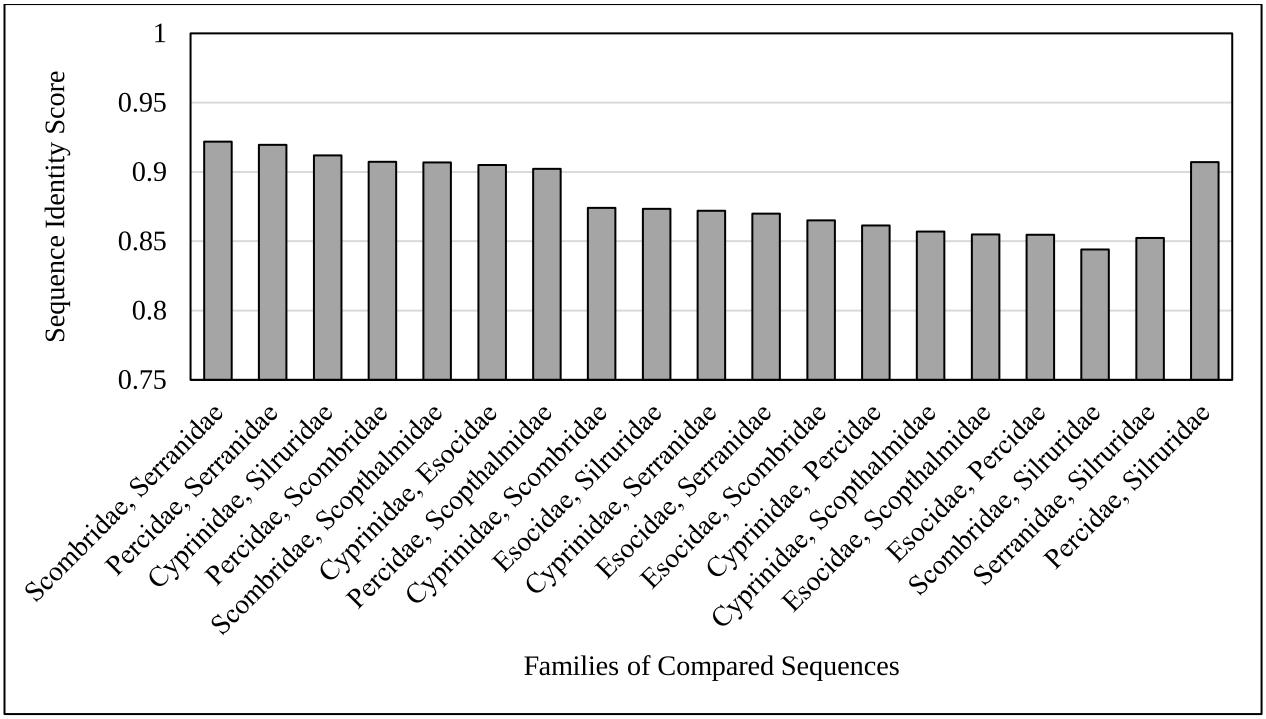


***Supplementary Figure S2 - Displaying the SIS values obtained through pairwise comparison of sequences drawn from an NCBI blast search. Sequences were selected where supporting PMF data was available within the datasets utilised in this study. Taxa information for each sequence is given to the family level.***

***Supplementary Table S1 - Breakdown of taxa from all combined datasets included in this study, including the number of individuals belonging to that family present in the dataset.***

| Family | Group | Number of individuals |
|---|---|---|
| Bovidae | Mammal | 10 |
| Canidae | Mammal | 10 |
| Elephantidae | Mammal | 10 |
| Equidae | Mammal | 10 |
| Leporidae | Mammal | 10 |
| Muridae | Mammal | 10 |
| Mustelidae | Mammal | 10 |
| Rhinocerotidae | Mammal | 10 |
| Ursidae | Mammal | 10 |
| Cyprinidae | Fish | 15 |
| Esocidae | Fish | 20 |
| Haemulidae | Fish | 7 |
| Labridae | Fish | 7 |

MOLECULAR ECOLOGY RESOURCES

| | | |
|---|---|---|
| Lutjanidae | Fish | 16 |
| Percidae | Fish | 18 |
| Scaridae | Fish | 35 |
| Scombridae | Fish | 24 |
| Scophthalmidae | Fish | 10 |
| Serranidae | Fish | 48 |
| Siluridae | Fish | 10 |
| Bufonidae | Amphibian | 10 |

***Supplementary Table S2 - Methods and parameters used in the R package 'MALDIQuant' to process ZooMS datasets, as described in Baker et al., (2023). *= method applied only for NEDMID analysis.***

| Function | Method | Parameters | Notes |
|---|---|---|---|
| trasnformIntensity* | SQRT | - | - |
| smoothIntensity* | SavitzkyGolay | HWS=6 | - |
| removeBaseline | TopHat | HWS=10 | - |
| calibrateIntensity* | TIC | | |
| alignSpectra* | lowess | HWS=20<br>SNR=2<br>Tolerance=0.002 | - |
| detectPeaks | MAD | HWS=20<br>SNR=4 | - |
| monoisotopic | - | chargeState=1.3<br>tolerance=1<br>intensityTolerance=1.0 | Reference table (Averagine table) required |
| binPeaks | Strict | tolerance=10 | Executed recursively until peak saturation |
| filterPeaks | - | minFrequency=0.01 | |
